# Revisiting Gender Differences in Somatic Symptoms of Depression: Much Ado about Nothing?

**DOI:** 10.1371/journal.pone.0032490

**Published:** 2012-02-24

**Authors:** Vanessa C. Delisle, Aaron T. Beck, Keith S. Dobson, David J. A. Dozois, Brett D. Thombs

**Affiliations:** 1 Department of Psychiatry, McGill University, Montréal, Québec, Canada; 2 Department of Epidemiology, Biostatistics and Occupational Health, McGill University, Montréal, Québec, Canada; 3 Department of Medicine, McGill University, Montréal, Québec, Canada; 4 School of Nursing, McGill University, Montréal, Québec, Canada; 5 Department of Psychiatry, Jewish General Hospital, Montréal, Québec, Canada; 6 Department of Psychiatry, University of Pennsylvania, Philadelphia, Pennsylvania, United States of America; 7 Department of Psychology, University of Calgary, Calgary, Alberta, Canada; 8 Department of Psychology, University of Western Ontario, London, Ontario, Canada; University of Sydney, Australia

## Abstract

**Background:**

Women have a higher prevalence of Major Depressive Disorder (MDD) and report more severe depressive symptoms than men. Several studies have suggested that gender differences in depression may occur because women report higher levels of somatic symptoms than men. Those studies, however, have not controlled or matched for non-somatic symptoms. The objective of this study was to examine if women report relatively more somatic symptoms than men matched on cognitive/affective symptoms.

**Methods:**

Male and female patients receiving treatment for MDD in outpatient psychiatric clinics in New Jersey and Pennsylvania, USA were matched on Beck Depression Inventory-II (BDI-II) cognitive/affective symptom scores. Male and female BDI-II somatic symptom scores were compared using independent samples 2-tailed *t*-tests.

**Results:**

Of 472 male and 1,026 female patients, there were 470 male patients (mean age = 40.1 years, SD = 15.1) and 470 female patients (mean age = 43.1 years, SD = 17.2) successfully matched on BDI-II cognitive/affective symptom scores. Somatic symptoms accounted for 35% of total BDI-II scores for male patients versus 38% for matched female patients. Female patients had somatic symptom scores on average 1.3 points higher than males (p<.001), equivalent to 4% of the total BDI-II scores of female patients. Only 5% of male patients and 7% of female patients scored 2 or higher on all BDI-II somatic symptom items.

**Conclusions:**

Gender differences in somatic scores were very small. Thus, differences in the experience and reporting of somatic symptoms would not likely explain gender differences in depression rates and symptom severity.

## Introduction

Women are 2–3 times more likely to experience Major Depressive Disorder (MDD) than men [1], [2] and have higher scores on self-report depression symptom measures [3], [4]. The higher prevalence of MDD among women compared to men begins in early adolescence and continues through adulthood [5], [6]. Similarly, women are more likely to be diagnosed with other psychiatric disorders that involve physical symptoms, including somatoform disorders, such as Somatization Disorder and Conversion Disorder [7]–[9]. Some of the symptoms of these disorders overlap substantially with symptoms of MDD. Women may also more frequently report other physical symptoms potentially related to emotional distress (e.g., headache, back pain) than men [10]–[12].

Many studies have examined whether men and women report somatic symptoms of depression differently or whether the number of somatic symptoms reported by men and women might explain gender differences in depression rates and severity [8], [13]–[26]. Since women have higher rates of depression than men, it would be expected that women would have higher cognitive/affective and somatic symptoms than men on average, but that the composition of symptoms among men and women would be similar. Otherwise, it would suggest that assessments of depression rates or severity may reflect different symptoms for men and women. Among people diagnosed with MDD, if there were more somatic symptoms among women, this would raise the possibility that increased reporting of somatic symptoms potentially unrelated to depression may influence the diagnosis of depression among women.

Two studies have used data from large US population surveys to examine whether there were gender differences in the rates of “somatic depression” among people with MDD [21], [22]. In those studies, “somatic depression,” was defined as having appetite disturbance, sleep disturbance and fatigue. The studies reported that women with MDD were twice as likely to endorse all three somatic symptoms versus men with MDD and therefore, that women with MDD were twice as likely to experience “somatic depression” than men with MDD. Men conversely, were slightly more likely than women to report a combination of cognitive/affective and somatic symptoms or only cognitive/affective symptoms. The conclusions of the studies suggested that differences in rates of “somatic depression” could explain higher rates of depression among women compared to men. However, in one of the studies, which used data from the National Comorbidity Survey, the absolute rate of “somatic depression” was only 8% for women with MDD and 4% for men with MDD [21]. In the other study, which combined data from the National Institute of Mental Health (NIMH) Collaborative Study on the Psychobiology of Depression and the Epidemiologic Catchment Area (ECA) Study, the absolute rates were 3% for women with MDD and 1.5% for men with MDD [22]. Thus, among people with MDD, somatic symptoms were more prominent among women compared to men. Notably, however, although the relative rate of “somatic depression” was larger for women, the proportion of depressed men and women with “somatic depression” was very small in both studies. Furthermore, the absolute difference in rates of “somatic depression” was very small. In addition, neither study controlled for the overall number of symptoms endorsed. As such, it is possible that the higher likelihood of endorsing all 3 somatic symptoms among women compared to men reflected a higher number of symptoms overall rather than increased somatic symptoms, specifically.

Many other studies have examined whether there are gender differences in the endorsement of individual depressive symptoms [8], [13]–[20], [23]–[26]. The results of those studies, however, have been inconsistent. Some studies have identified some somatic symptoms more frequently reported by women, but not the same somatic symptoms across studies [8], [14]–[20], [23]–[26]. On the other hand, a number of studies have identified cognitive/affective symptoms more frequently reported by women than men [8], [13], [15], [23], [24].

Thus, it is not clear whether and to what degree women report higher levels of somatic symptoms than men with similar levels of cognitive/affective symptoms. MDD is diagnosed per the DSM-IV [9] based on the presence of at least 5 of 9 symptoms, including 1 of 2 core symptoms of depressed mood or anhedonia. For the purpose of evaluating the relative somatic symptom burden of women and men with MDD, however, continuous measures, such as the Beck Depression Inventory-II (BDI-II) [27] may be advantageous compared to simply counting symptoms. The reason for this is that continuous measures assess a wider range of symptom experiences and provide continuous scores that allow parsing of variance into somatic and cognitive/affective components. The objective of this study was to determine whether women with MDD report higher levels of somatic symptoms on the BDI-II than men with MDD matched on cognitive/affective BDI-II symptoms.

## Methods

### Ethics Statement

This study was a secondary analysis of de-identified data from studies that were originally approved by the institutional review boards of the University of Medicine and Dentistry of New Jersey and the University of Pennsylvania. All patients provided written consent for their information to be stored in hospital databases and used for research.

### Participants and Procedure

The sample consisted of psychiatry outpatients (≥18 years old) with a primary DSM-IV diagnosis of MDD. Patients were evaluated and diagnosed by experienced psychiatrists or psychologists at an outpatient psychiatric clinic associated with the Department of Psychiatry, University of Medicine and Dentistry of New Jersey or at the Beck Institute for Cognitive Therapy and Research in Pennsylvania, USA. Patients completed the BDI-II at intake, prior to beginning treatment, as part of a standard battery of psychological tests. Patient participation was voluntary and informed consent was obtained before the instruments were administered.

Male and female patients who completed all BDI-II items were matched on cognitive/affective symptom scores. To do this, the number of male and female patients with each possible cognitive/affective score was compared. In order to have the same number of male and female patients at each cognitive/affective score level, we included all patients at each cognitive/affective score level from the group (male or female) with the smaller number of patients. Then the same number of patients from the other group with that cognitive/affective score was randomly selected. To do this, we generated a random number for each patient using SPSS (normal distribution, mean = 0, standard deviation = 1). We then selected the appropriate number of patients from the larger group based on random numbers, with the patients with the highest random numbers selected. For example, if 10 male patients and 15 female patients had cognitive/affective scores of 5, then all 10 male patients were selected along with the 10 female patients with the highest random numbers of the 15 female patients. Patients who were not matched were not included in the primary analyses.

### Measure

Symptoms of depression were assessed using the 21-item BDI-II [27]. BDI-II items consist of four statements, scored 0–3, with higher scores indicating increasing symptom severity. Respondents are instructed to describe the way they have been feeling during the past two weeks. There is extensive evidence of the validity and reliability of the BDI-II in both psychiatric and non-psychiatric populations [28], [29]. Based on reviews of existing factor models [30], [31] and item content, scores on BDI-II items 1–14 (*sadness, pessimism, past failure, loss of pleasure, guilty feelings, punishment feelings, self-dislike, self-criticalness, suicidal ideation, crying, agitation, loss of interest, indecisiveness, worthlessness*) were summed to calculate cognitive/affective symptom scores. Items 15–21 (*loss of energy, sleep problems, irritability, appetite problems, concentration, fatigue, sexual disinterest*) were summed to calculate somatic symptom scores.

### Analysis of the Data

Independent samples 2-tailed *t*-tests were used to test whether matched male and female patients differed with regard to BDI-II somatic symptom scores overall. Standardized mean differences were calculated using the Hedges's *g* statistic [32]. Male and female patients' somatic symptom item scores were similarly compared. Hochberg's Sequential Method was used to maintain a family-wise Type I error rate of α<.05 for multiple comparisons [33]. Two sensitivity analyses were also conducted. First, matched male and female patients were compared using ANCOVA to adjust for age. Second, to supplement the main analysis, we analyzed data from all patients, rather than just matched patients, by using linear regression to assess the difference on somatic symptom scores between male and female patients, controlling for BDI-II cognitive/affective symptom scores.

In addition, similar to Silverstein's analyses of rates of male and female patients with MDD who endorsed all somatic symptoms, patients who scored 2 or above on all seven BDI-II somatic symptom items were classified as exhibiting “somatic depression.” The χ^2^ statistic was used to compare the prevalence of “somatic depression” between male and female patients.

## Results

### Sample Characteristics

Prior to matching, mean BDI-II scores were 27.5 (SD = 11.5) for 472 male patients and 29.8 (SD = 12.0) for 1,026 female patients. There were 470 exact matches using cognitive/affective symptom scores, and matched females were on average 3 years older than matched males (43.1 years, SD = 17.2 versus 40.1 years, SD = 15.1, respectively, p = .005). The majority of male (89%) and female (90%) patients were White.

### BDI-II Somatic Symptom Scores

Post-match mean BDI-II scores were 27.5 (SD = 11.5) for 470 male patients and 28.7 (SD = 11.7) for 470 female patients. As shown in Table 1, somatic symptom scores were 1.3 (95% confidence interval [CI] = 0.7 to 1.8) points higher among female than male patients, equivalent to 4% of the mean total BDI-II score of female patients (p<.001). Somatic symptoms accounted for 35% of total BDI-II scores for male patients versus 38% for female patients, a negligible raw difference of 3%. Hedges's *g* for the difference in somatic scores between male and female patients was 0.30, which is a relatively small difference based on Cohen's operational definitions (small = 0.2, medium = 0.5, large = 0.8) [34]. The results for all the analyses were unchanged when controlling for age (somatic symptom score difference = 1.3, 95% CI = 0.8 to 1.8, p<.001). Results were also similar when somatic symptom scores were regressed on sex and cognitive/affective symptom scores (somatic symptom score difference = 1.2, 95% CI = 0.8 to 1.5, p<.001) for all patients, rather than just matched patients. As shown in Table 2, the higher somatic scores among female patients were due to significantly higher scores on items 15 (*loss of energy*), 18 (*appetite problems*), 20 (*tiredness or fatigue*), and 21 (*sexual disinterest*).

**Table 1:** Comparison of BDI-II Somatic Symptom Scores for Female Patients Versus Male Patients.

N Per Group	Mean Cognitive/Affective Scores^1^: Females and Males	Mean Somatic Scores^1^: Females (% Total Scores)	Mean Somatic Scores^1^: Males (% Total Scores)	Difference in Somatic Scores^1^ (% Female Scores)	P-Value
470	17.95	10.79 (37.5)	9.52 (34.7)	1.27 (4.4)	<.001

BDI-II = Beck Depression Inventory-II.

1 Definitions of cognitive/affective and somatic scores in text.

**Table 2:** Comparison of BDI-II Somatic Symptom Item Scores for Female Patients Versus Male Patients.

BDI-II Item	Females (Mean/SD)	Males (Mean/SD)	Difference in Somatic Item Scores^1^ (% Female Item Scores)	P-Values
15. Loss of Energy	1.69/0.87	1.45/0.80	0.24 (14.2)	<.001^2^
16. Sleep Problems	1.63/0.99	1.63/0.89	0.00 (0.0)	.910
17. Irritability	1.32/1.01	1.29/0.97	0.03 (2.3)	.674
18. Appetite Problems	1.41/0.01	1.13/0.95	0.28 (19.9)	<.001^2^
19. Concentration	1.59/0.87	1.51/0.86	0.08 (5.0)	.178
20. Tiredness or Fatigue	1.72/0.94	1.47/0.94	0.25 (14.5)	<.001^2^
21. Sexual Disinterest	1.43/1.17	1.04/1.01	0.39 (27.3)	<.001^2^

BDI-II = Beck Depression Inventory-II.

1 Definition of somatic scores in text.

2 Statistically significant based on Hochberg's Sequential Method.

### BDI-II Somatic Depression

Overall, 5% (25 of 470) of male patients and 7% (34 of 470) of female patients scored 2 or above on all seven BDI-II somatic symptom items. There was no statistically significant difference between male and female patients with regard to the prevalence of “somatic depression” based on scoring 2 or above on all seven BDI-II somatic symptom items (*loss of energy, sleep problems, irritability, appetite problems, concentration, fatigue, sexual disinterest*; p = .226).

## Discussion

The main finding of this study was that BDI-II somatic symptom scores were only minimally higher for female versus male psychiatry outpatients with MDD matched on cognitive/affective symptom scores. Women scored just over a point higher than men, accounting for only 4% of their total BDI-II scores. Furthermore, based on BDI-II somatic symptoms item scores, only 7% of women versus 5% of men and were classified as having “somatic depression.”

These results, numerically, are consistent with results from two prominent earlier studies that reported that “somatic depression” was twice as prevalent among women with MDD compared to men with MDD [21], [22]. In those two studies, rates of “somatic depression” were 8% and 3% for women versus 4% and 1.5% for men, respectively. In both of those studies, and in the present study, however, more than 90% of men and women with MDD were not classified as having “somatic depression.”

The key difference between the present study and the two earlier studies by Silverstein [21], [22] is that the previous studies focused on relative risk without interpreting the meaningfulness of findings in terms of absolute risk. In the context of very low rates, however, communication of relative risk is often highly misleading [35]–[38]. For instance, increasing risk from 1 in 10,000 to 2 in 10,000 would technically be “double the relative risk,” but, in most cases, would not be clinically meaningful. Silverstein suggested that the increased relative risk of “somatic depression” for women was a possible explanation for gender differences in depression rates and severity, but only a very small percentage of MDD patients were classified as having “somatic depression,” which was also the case in the current study. Thus, the very minor differences between rates of “somatic depression” for men and women in all of these studies, along with the minimal gender difference in BDI-II somatic symptom scores in the present study, suggest that gender differences in depression are not adequately explained in terms of somatic symptoms.

A number of other studies have examined whether there are gender differences in the endorsement of depressive symptoms on an item-by-item basis [8], [13]–[20], [23]–[26]. Some studies have found that women are more likely to report certain somatic items, including appetite disturbances, weight disturbances and fatigue than men [8], [14]–[20], [23]–[26]. However, the specific somatic symptoms identified as potentially different for men and women are not the same across studies, and most somatic symptom items analyzed show no differences. On the other hand, some studies have reported that women are more likely than men to report certain cognitive/affective items, such as tearfulness, feelings of guilt, feelings of worthlessness, sexual disinterest and thoughts of death, than men [8], [13], [15], [23], [24]. However, as with somatic symptoms, consistent patterns do not emerge across studies and, for most cognitive/affective symptoms examined, there are no differences. Possible reasons that have been suggested to explain these findings include the use of different methods to assess symptoms of depression and different patients and settings [19]. In addition, all of these studies conducted statistical tests on many different items, without adjustment for multiple comparisons, and based their conclusions of gender differences on differences in small numbers of items among many tested. Given this, the inconsistent findings would be consistent with very minimal differences overall, as we found in the present study. In the present study, although we presented item-by-item data, our hypothesis was tested based on a single comparison of differences in overall somatic symptom scores.

There is no debating that women have higher rates of depression and report more severe depressive symptoms than men, and it is important to better understand how and why these gender differences in depression occur in order to create more efficacious treatments. The findings of this study, however, do not support the existence of a somatic subtype of depression among women that may explain gender differences in depression. Given this finding, other explanations that have been advanced may be more promising. For instance, it has been suggested that gender differences in depression may be related to 1) prior anxiety, which is more common among women than men [39], 2) the willingness of women to admit their depression more than men [40], 3) the tendency for women to ruminate about problems more than men [41], 4) higher levels of stress and lower levels of fulfillment associated with traditional male versus female sex roles [42], and 5) hormonal changes during puberty [43].

One limitation of this study is that we studied a sample of adults with MDD who sought treatment at one of two clinics in New Jersey or Pennsylvania, USA, which could limit the generalizability of results. On the other hand, the results of the study are generally similar to Silverstein's [21], [22] previous findings using larger, more representative samples of patients, and robustly demonstrate that somatic symptom reporting is not likely an explanation for gender differences. A second limitation of this study is that, as in Silverstein's studies [21], [22], our definition of “somatic depression” was arbitrary. However, the amount of men and women with “somatic depression” in this study was similar to that in Silverstein's studies [21], [22] and facilitated comparisons. Also, since different assessment methods were used, the definitions of “somatic depression” were somewhat different. We used a definition that was based on published factor analyses of the BDI-II, which may differ somewhat from other conceptualizations. A third limitation of this study is that we did not have information regarding whether or not patients were diagnosed with additional psychiatric diagnoses besides MDD and therefore, it is possible that this sample may not be representative of patients only with MDD. Lastly, other possible limitations of the study were that MDD diagnoses were not based on a structured clinical interview and that depressive symptoms were assessed using the BDI-II, which is not a diagnostic tool. The BDI-II, however, does contain the core symptoms that are covered in the DSM-IV. In addition, using the BDI-II allowed for an assessment of continuous symptom scores and overall contribution of somatic symptoms whereas a structured clinical interview only permits counting the presence or absence of symptoms.

In summary, it is well-known that women have a higher prevalence of MDD and report more severe depressive symptoms than men. The results of this study show that differences in the experience and reporting of somatic symptoms explain only a small portion of these gender differences. Future studies examining this issue should focus on additional explanations.
